# Evaluation of the Relationship between Drink Intake and Periodontitis Using KoGES Data

**DOI:** 10.1155/2021/5545620

**Published:** 2021-03-16

**Authors:** Seok Jin Hong, Bin Kwon, Byoung Eun Yang, Hyo Geun Choi, Soo Hwan Byun

**Affiliations:** ^1^Research Center of Clinical Dentistry, Hallym University Clinical Dentistry Graduate School, Chuncheon 24252, Republic of Korea; ^2^Department of Otorhinolaryngology-Head & Neck Surgery, Dongtan Sacred Heart Hospital, Hallym University College of Medicine, Dongtan 18450, Republic of Korea; ^3^Department of Oral & Maxillofacial Surgery, Dentistry, Sacred Heart Hospital, Hallym University College of Medicine, Anyang 14068, Republic of Korea; ^4^Hallym Data Science Laboratory, Hallym University College of Medicine, Anyang 14068, Republic of Korea; ^5^Department of Otorhinolaryngology-Head & Neck Surgery, Sacred Heart Hospital, Hallym University College of Medicine, Anyang 14068, Republic of Korea

## Abstract

It was hypothesized that periodontal diseases could be influenced by nutrition and food types. However, the role of nutritional factors in the risk of periodontal disease has not been clearly elucidated. This study was aimed at investigating the relationship between coffee, green tea, or soft drink intake and periodontitis. This prospective cohort study used epidemiological data from 2004 to 2016 from the Korean Genome and Epidemiology Study. Among 173,209 participants, 9,933 with periodontitis and 124,922 controls were selected. The frequency histories of coffee/green tea/soft drink intake among the participants were analyzed, and intake was categorized as no drink, mild drink (one time a month through six times a week), and heavy drink (one or more times a day). Variable factors were adjusted using logistic regression analysis (adjusted model). The chi-square test and independent *t*-test were used for statistical analysis. Adjusted odds ratio (aOR) for coffee or green tea intake and periodontitis were not statistically significant. The aOR was 1.16 (95% confidence interval (CI) = 1.11–1.21, *P* < 0.001) for mild soft drink intake and 1.02 (95%CI = 0.96–1.09, *P* = 0.518) for heavy soft drink intake. Subgroup analysis showed that mild soft drink intake was significant across all groups (*P* < 0.05), whereas coffee and green tea intakes were not significant in any subgroup. Overall, the study elucidated an association between mild soft drink intake and periodontitis.

## 1. Introduction

Periodontal diseases are highly prevalent, affecting approximately 538 million people worldwide [1, 2]. Periodontitis is a chronic inflammatory disease, aggravated by microorganisms in the periodontal pocket, tooth surface, and periodontium [3]. The most important causative factors of periodontal pathology are related to improper compliance with oral hygiene. Maintenance of effective plaque control is the cornerstone of any attempt to prevent and control periodontal disease [4]. After approximately 50 years of experimental research in various geographical and social settings, it was confirmed that effective removal of dental plaque is essential for dental and periodontal health [5].

These inflammatory disease could be worsened by multiple factors such as systemic disease, nutrients, social factors, obesity, and genetic factors [6]. Periodontal diseases are affected by nutrition and food types. Conversely, they could decrease the quality of life due to difficulty in mastication and proper intake of nutrients [7].

The role of nutritional factors in the risk of periodontal disease has not been clearly elucidated. Nowadays, several nutrients and foods are of interest in the prevention of dental diseases. In this study, we focus on some of them.

First, green tea has received attention based on many reports that showed its effects on periodontal health [8]. The main ingredients of green tea are catechins. Catechins can be divided into four groups: epigallocatechin-3-gallate (EGCG, 59%), epigallocatechin (EGC, 19%), epicatechin-3-gallate (ECG, 13.6%), and epicatechin (EC, 6.4%) [9]. In addition, green tea contains antioxidant-based components such as vitamins A/C/E [8]. Green tea constitutes powdered ground green leaves and can cause staining of the teeth. Many patients have been worried that the staining of teeth or residue after drinking green tea can lead to caries and periodontal plaques. However, previous studies have shown that the intake of green tea has a positive effect on periodontal disease [10, 11]. When a green tea extract was applied directly to the periodontal tissue, it showed a beneficial effect on periodontal health [12].

Second, coffee has been assumed to be related to many diseases, and coffee intake has been linked to adverse effects [13]. Coffee is a caffeine-based beverage commonly consumed worldwide, and it currently ranks first among South Korea's most frequently consumed beverage items. Daily coffee consumption is thought to range from 140 to 434 mL [14]. A study showed that the intake of fat ingredients used in coffee creamers and sugar could contribute to systemic and metabolic diseases [15]. Another study reported that coffee consumption might be an independent risk indicator of periodontal disease in Korean adults [14]. One study also showed that chlorogenic acid in coffee has antimicrobial activity and reduces the protease activity of *Porphyromonas gingivalis* [16]. *Porphyromonas gingivalis* is one of the bacteria implicated in bacterial plaque biofilms, and it contributes to the progression of periodontal disease [17].

Lastly, periodontal diseases have also been associated with the consumption of soft drinks, which are carbonated sugar-sweetened beverages. These beverages are the main source of excessive calorie intake [18]. The high consumption of soft drinks has been associated with systemic problems such as diabetes, obesity, fatty liver, metabolic syndrome, and cardiovascular disease [19]. A previous study reported that high soft drink consumption was related to a higher number of teeth with deep periodontal pockets and bleeding on probing in pregnant women [20]. The study suggested that soft drink consumption could be a risk factor for systemic inflammation associated with periodontal disease [20]. However, this study had a small number of participants (*n* = 1185) and used very few adjusting factors for surveillance bias reduction. Fann et al. showed that carbonated drink was a risk factor for periodontitis with chronic inflammation due to the high glycemic level [21]. The study suggested that the periodontal condition could be ameliorated by lowering carbonated drink consumption [21]. This study recruited subjects from 2005 to 2009 and examined a relatively small number of participants (*n* = 10,213). Another study reported that the consumption of carbonated beverages was positively associated with the risk of periodontal disease in Korean adults [22]. Similar to previous studies, this study also had a relatively small number of participants (*n* = 5,517) recruited between 2008 and 2010 with consideration of a few confounding factors [22].

The objective of this study was to investigate the relationship between coffee, green tea, or soft drink (carbonated sugar-sweetened beverages) intake and periodontitis using data from the Korean Genome and Epidemiology Study (KoGES). This study implemented a large population dataset with adjustment for various influential factors.

## 2. Materials and Methods

### 2.1. Study Population and Data Collection

This prospective cohort study used data from 2004 through 2016 from the KoGES. A detailed description of the data has been provided in a previous study [23]. The study was approved by the Institutional Review Board of Hallym University Sacred Heart Hospital. The requirement for written informed consent was waived by the Institutional Review Board (2019-02-020). Among the KoGES Consortium, we used the KoGES health examinee (HEXA) data consisting of urban residence participants ≥ 40 years old. It consisted of baseline data for the period 2004–2013 and follow-up data for the period 2012–2016.

### 2.2. Participant Selection

**Figure 1:**
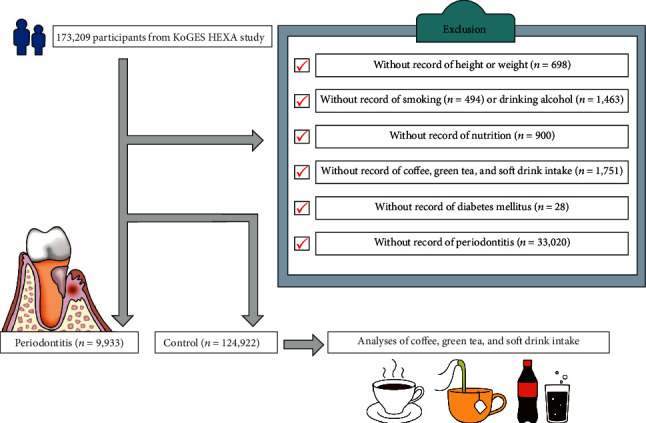
Schematic illustration of the participant selection process used in the present study. Of a total of 173,209 participants, 9,933 had periodontitis and 124,922 did not (controls).

Of the 173,209 participants, we excluded those with the following missing records: height or weight (*n* = 698), smoking history (*n* = 494), alcohol-drinking habits (*n* = 1,463), nutrition information (*n* = 900), coffee/green tea/soft drink intake (*n* = 1,751), diabetes mellitus history (*n* = 28), and periodontitis history (*n* = 33,020). Many participants were excluded due to periodontitis histories in that they were not surveyed for periodontitis in the KoGES from 2004 through 2006. Finally, 9,933 patients with periodontitis and 124,922 control participants (nonperiodontitis) were selected (Figure 1). Then, we analyzed the frequency of coffee/green tea/soft drink intake.

### 2.3. Survey

The participants were asked for their previous history of periodontitis and diabetes mellitus by trained interviewers. The questions were formulated, such as “Have you ever been diagnosed with periodontitis?” The questions were to be answered with a simple “yes” or “no.” Body mass index (BMI) was calculated using the health checkup data and expressed in kilogram per square meter. Based on the smoking history, participants were categorized as a nonsmoker (<100 cigarettes in entire life), past smoker (quitted more than one year ago), and current smoker. Participants with alcohol-drinking habits were categorized as a nondrinker, past drinker, and current drinker. Their nutritional intake (total calories (kcal/day); protein (g/day), fat (g/day); and carbohydrate (g/day)) was surveyed using a food-frequency questionnaire, which was validated in a previous study [24]. The income groups were categorized as nonrespondent, low income (~<$2,000 per month), middle income (~$2,000–$3,999 per month), and high income (~≥$4,000 per month) based on their household income.

The frequency of coffee/green tea/soft drink intake was categorized as no drink, one time a month, two to three times a month, one to two times a week, three to four times a week, five to six times a week, one to two times a day, three to four times a day, and five or more times a day. As many participants were in the one to two times a day group, we finally classified them into three groups: no drink, mild drink (one time a month through six times a week), and heavy drink (one or more times a day).

### 2.4. Statistical Analyses

We compared the general characteristics of the periodontitis and control participants. Chi-square tests were used to compare the sex, income group, smoking, drinking and alcohol consumption, and frequency and amount of coffee/green tea/soft drink intake among the groups. Independent *t*-tests were used to compare age, BMI, and nutritional intake [25, 26]. Odds ratios (ORs) were calculated to compare the association between coffee, green tea, and soft drink intake and periodontitis in this cross-sectional study. To analyze the OR of coffee, green tea, and soft drink intake for periodontitis, crude and adjusted ORs (model 1 (adjusted for age, sex, BMI, diabetes mellitus, smoking, alcohol consumption, and nutritional intake) and model 2 (model 1 plus coffee, green tea, and soft drinks)) were calculated. For the subgroup analyses, the effects of age and sex groups were examined and stratified based on the overall median age (≤52 years old and >52 years old). Two-tailed analyses were conducted, and *P* values < 0.05 were considered to indicate statistical significance [27–29]. The results were statistically analyzed using SPSS v. 24.0 (IBM, Armonk, NY, USA).

## 3. Results

There were variations in the general characteristics between participants with periodontitis and the control participants (Table 1).

Coffee, green tea, and soft drink intakes were related to periodontitis in model 1 after adjusting for age, sex, BMI, diabetes mellitus, smoking, alcohol consumption, and nutritional intake (Table 2).

The adjusted OR (aOR) for coffee and green tea intake for periodontitis did not reach statistical significance in model 2, while that for mild soft drink was 1.16 (95% confidence interval (CI) = 1.11–1.21, *P* < 0.001) and that for heavy soft drink was 1.02 (95%CI = 0.96–1.09, *P* = 0.518) (Table 2).

In the subgroup analyses, the aOR of mild soft drink showed a significant difference in all groups. The aORs were 1.20 (95%CI = 1.12–1.29) in age ≤ 52 years old; 1.13 (95%CI = 1.07–1.20) in >52 years old; 1.26 (95%CI = 1.17–1.36) in men; and 1.10 (95%CI = 1.03–1.16) in women (each with *P* < 0.05, Table 3). However, the aORs of heavy soft drink did not show any significant difference in any of the subgroups.

The aORs of coffee and green tea intake did not show significant differences in any of the subgroups (Table S1).

## 4. Discussion

This study was based on the hypothesis that coffee/green tea/soft drink intake was associated with periodontitis. This study investigated the relationship between coffee, green tea, or soft drink (carbonated sugar-sweetened beverages) intake and periodontitis using data from the KoGES. However, coffee and green tea intake by patients with periodontitis did not show any statistically significant difference in model 2 after adjusting for soft drinks. Additionally, heavy consumption of soft drink did not show any statistically significant difference in any of the subgroups. Only mild consumption of soft drinks showed statistically significant difference in all the subgroups. These results are quite different from those obtained from other studies.

First, there have been very few studies on the relationship between soft drinks and periodontal health [20–22]. Fann et al. reported a frequency-dependent relationship between the intake of soft drinks and periodontal disease in Taiwanese middle-aged adults. Song et al. showed that consumption of soft drinks was related to the risk of periodontal disease in Korean adults [22]. In particular, by reducing the intake of soft drinks, the periodontal condition in nonobese Koreans could be ameliorated [22]. Most studies asserted that the consumption of soft drinks was associated with the risk of periodontitis. However, the present study revealed that only mild soft drinks showed a significant difference in all the subgroups, and heavy soft drinks did not show any significant difference in any of the subgroups. This result proves the relationship between soft drinks and periodontal health but does not indicate a frequency-dependent relationship. This phenomenon could be explained by other perspectives. A study reported that individuals with periodontitis avoided soft drinks, sweets, cold drinks, and cold food more often than that without [30]. In other words, individuals who consumed soft drinks frequently and heavily may not have periodontitis. Soft drinks in Korea are mostly cold and consumed with ice. For this reason, individuals with periodontitis might not consume cold soft drinks frequently. As a result, mild soft drinks showed statistical significance in all subgroups, possibly due to the interaction between soft drinks and periodontitis (Table 2). Mechanisms linking soft drink intake and periodontal disease could involve the separate or combined effects of oxidative stress or bone mineralization [31–37]. Associations between heavy soft drink intake and periodontitis may be due to the induction of inflammatory pathways or metabolic syndrome resulting from excess sugar consumption, which has been previously associated with periodontal disease [20, 38–40]. However, further studies are needed to verify these potential mechanisms more precisely [21].

Second, many studies have shown that the catechin component of green tea has a beneficial effect on periodontal health [9]. Gartenmann et al. reported that the local application of catechin as a supplement to scaling and root planing might induce reductions in probing depth [41]. Catanzaro et al. reported that daily intake of green tea has a therapeutic effect on diabetic vascular disorders in the periodontal ligament and aggravation of periodontitis due to long-term hyperglycemia in type 1 diabetic rats [42]. Results of another study showed that daily green tea intake might positively influence treatment effects for periodontal disease and green tea extract may be applied as an adjunct to phase one periodontal therapy, which includes scaling and root planing [43]. In summary, these previous studies showed that green tea has a positive effect on periodontal health.

This study analyzed the association between green tea intake and periodontitis after adjustment for soft drinks. Green tea intake was related to periodontitis in model 1 before adjusting for soft drinks but showed no association in model 2 after this adjustment (Table 2). This result revealed that adjusting for soft drinks is essential to evaluate the association between periodontitis and green tea intake.

Finally, there have been controversial opinions about the effects of coffee on periodontal health. Tanaka et al. reported that the daily intake of black coffee had a negative effect on alveolar bone regeneration [44]. Han et al. reported that coffee intake might be an independent risk factor for periodontal disease in Korean male adults and suggested that the periodontal condition may improve with a reduction in coffee intake [14]. In contrast, a cross-sectional study conducted by Machida et al. showed an inverse association between coffee intake and severe periodontitis in patients during the maintenance period of periodontal treatment [45]. Another study conducted by Ng et al. indicated that a beneficial association was noted between higher intake of coffee and periodontal health. The results of the study indicated that high consumption of coffee and caffeine was not harmful to periodontal health [46].

In addition, previous studies on coffee did not perform any adjustment for the soft drinks. In fact, this study also analyzed the data after adjusting for coffee and green tea but not for soft drinks. The analysis showed a significant association between coffee and periodontitis. There were statistically significant differences in heavy coffee consumption and moderate green tea consumption (Table S1). However, coffee was related to periodontitis in model 1 before adjusting for soft drinks but showed no association with periodontitis in model 2 after adjusting for soft drinks (Table 2). Generally, individuals who like coffee or green tea may also like soft drinks; hence, the adjustment for soft drinks would be helpful to obtain more accurate results [47, 48]. The findings of the present study are also adjusted for nutritional intake, age, sex, obesity, income, smoking, diabetes, and alcohol consumption (Table 1). This approach provides the major differences between the present study and previous studies.

### 4.1. Limitations

This study used a large pool of data; nevertheless, there are some limitations. First, it was impossible to adjust for all the factors that were not included in the original data since the data from the KoGES did not include all the confounding factors. For example, plaque control and sugar composition of soft drinks could be influential factors; however, they were not recorded and consequently could not be adjusted for in this study. In addition, while BMI may be a useful metric, it may not accurately reflect the overall body composition, such as the total proportion of body fat. We tried to adjust for as many factors as possible to minimize surveillance bias. Second, these data were collected using an individual questionnaire survey; hence, the precision of the data used in this study could be low. Third, the frequency of consumption could be a simple approach, since the volume of each intake and quality of the beverage may be different and may have an important influence on the analysis. Fourth, oral hygiene practices, such as brushing frequency, use of interdental cleaning devices, and dental check-ups, were not included in this study. However, it may still be practical to collect a large amount of data related to periodontitis and nutritional intake. Most nutritional studies have been based on questionnaires since surveyors are unable to directly check or follow the dietary intake and activities of study participants [21]. Moreover, most nutritional studies were not conducted in conjunction with a simultaneous oral examination. In addition, the validity of the questionnaires in terms of the frequency of smoking, alcohol consumption, and food intake is unclear. To collect exact data, the reliability and validity of the questionnaire survey should be examined in future studies. Lastly, the interaction model was not included in the analysis since we were unable to identify meaningful interactions between green tea, coffee, and soft drinks.

### 4.2. Strengths

Nevertheless, our findings provide valuable information about periodontitis and coffee/green tea/soft drinks. First, our study was performed on a large sample of the Korean population. Second, we considered nutritional intake, age, sex, obesity, income, smoking, diabetes, and alcohol consumption as factors to be adjusted in order to evaluate the independent relationship between periodontitis and coffee/green tea/soft drinks. Finally, the intake of soft drinks was adjusted in order to prove the association between periodontitis and coffee/green tea.

Many patients inquire clinicians regarding the relationship between drink intake and oral health; however, there was no conclusive evidence on this relationship. This study could provide the ground for a satisfactory explanation to the general public as it provides evidence regarding this association.

## 5. Conclusions

This study demonstrated that low-frequency soft drink intake could be associated with periodontitis. These findings emphasize the need for clinicians to examine dietary habits as an essential procedure for optimal oral hygiene care and periodontal treatment. Further, it is recommended that dentists should provide instructions for improvement in dietary habits in consultation with a dietitian.

## Figures and Tables

**Table 1:** General characteristics of the participants.

Characteristics	Total participants		*P* value
	Periodontitis	Control	
Total number (*n*, %)	9,933 (100.0)	124,922 (100.0)	
Age (mean, SD, y)	54.8 (7.9)	53.0 (8.3)	<0.001^∗^^†^
Sex (*n*, %)			<0.001^∗^^†^
Male	3,840 (38.7)	43,283 (34.6)	
Female	6,093 (61.3)	81,639 (65.4)	
BMI (mean, SD, kg/m^2^)	24.0 (2.9)	23.9 (2.9)	<0.001^∗^^†^
Income (*n*, %)			<0.001^∗^^†^
Missing, no response	757 (7.6)	10,784 (8.6)	
Lowest	3,424 (34.5)	35,464 (28.4)	
Middle	3,663 (36.9)	49,300 (39.5)	
Highest	2,089 (21.0)	29,374 (23.5)	
Smoking status (*n*, %)			<0.001^∗^^†^
Nonsmoker	6,658 (67.0)	90,822 (72.7)	
Past smoker	1,790 (18.0)	18,540 (14.8)	
Current smoker	1,485 (15.0)	15,560 (12.5)	
Diabetes mellitus (*n*, %)	1,171 (11.8)	9,789 (7.8)	<0.001^∗^^†^
Alcohol consumption (*n*, %)			<0.001^∗^^†^
Nondrinker	4,762 (47.9)	63,831 (51.1)	
Past drinker	477 (4.8)	4,521 (3.6)	
Current drinker	4,694 (47.3)	56,570 (45.3)	
Nutritional intake (*n*, %)			
Total calories (kcal/d)	1760.5 (579.9)	1749.2 (568.7)	0.056
Protein (g/d)	58.9 (26.6)	59.7 (26.4)	0.004^†^
Fat (g/d)	27.5 (18.5)	28.3 (18.2)	<0.001^∗^^†^
Carbohydrate (g/d)	315.0 (95.1)	309.8 (92.8)	<0.001^∗^^†^
Frequency of coffee (*n*, %)			<0.001^∗^^†^
No drink	1,613 (16.2)	20,160 (16.1)	
Mild	2,199 (22.1)	25,286 (20.2)	
Heavy	6,121 (61.6)	79,476 (63.6)	
Frequency of green tea (*n*, %)			<0.001^∗^^†^
No drink	4,133 (41.6)	53,638 (42.9)	
Mild	1,696 (17.1)	19,287 (15.4)	
Heavy	4,104 (41.3)	51,997 (41.6)	
Frequency of soft drink (*n*, %)			<0.001^∗^^†^
No drink	4,085 (41.1)	53,413 (42.8)	
Mild	4,415 (44.4)	51,710 (41.4)	
Heavy	1,433 (14.4)	19,799 (15.8)	

D: day; W: week; M: month; *n*: number; SD: standard deviation; BMI: body mass index. ^∗^*P* < 0.05 was considered statistically significant. ^†^Independent *t*-test or chi-square test.

**Table 2:** Crude and adjusted odds ratios (95% confidence interval) of coffee, green tea, and soft drink intake for periodontitis.

Characteristics	Odds ratios for periodontitis					
	Crude^‡^	*P* value	Model 1^‡,§^	*P* value	Model 2^‡,§^	*P* value
Total participants (*n* = 134,855)						
Coffee						
No drink	1.00		1.00		1.00	
Mild	1.09 (1.02–1.16)	0.015^∗^^†^	1.11 (1.04–1.19)	0.002^∗^^†^	1.04 (0.96–1.13)	0.379
Heavy	0.96 (0.91–1.02)	0.190	0.97 (0.92–1.03)	0.356	0.96 (0.89–1.02)	0.199
Green tea						
No drink	1.00		1.00		1.00	
Mild	1.14 (1.08–1.21)	<0.001^∗^	1.14 (1.07–1.20)	<0.001^∗^^†^	1.07 (0.96–1.13)	0.379
Heavy	1.02 (0.98–1.07)	0.294	0.99 (0.95–1.04)	0.694	0.96 (0.89–1.02)	0.199
Soft drink						
No drink	1.00		1.00		1.00	
Mild	1.12 (1.07–1.17)	<0.001^∗^^†^	1.17 (1.12–1.23)	<0.001^∗^^†^	1.16 (1.11–1.21)	<0.001^∗^^†^
Heavy	0.95 (0.89–1.01)	0.083	1.02 (0.95–1.08)	0.630	1.02 (0.96–1.09)	0.518

^∗^ *P* < 0.05 was considered statistically significant. ^†^Logistic regression model. ^‡^Model 1 was adjusted for age, sex, body mass index (BMI), diabetes mellitus, smoking, alcohol consumption, and nutritional intake. ^§^Model 2 was adjusted for model 1 plus frequency of coffee, green tea, and soft drink.

**Table 3:** Crude and adjusted odds ratios (95% confidence interval) of soft drink intake for periodontitis according to age and sex.

Characteristics	Odds ratios for periodontitis					
	Crude^‡^	*P* value	Model 1^‡,§^	*P* value	Model 2^‡,§^	*P* value
Age ≤ 52 years old (*n* = 66,737)						
No drink	1.00		1.00		1.00	
Mild	1.18 (1.10–1.26)	<0.001^∗^	1.21 (1.12–1.29)	<0.001^∗^	1.20 (1.12–1.29)	<0.001^∗^
Heavy	1.01 (0.92–1.11)	0.854	1.03 (0.94–1.14)	0.512	1.04 (0.94–1.14)	0.470
Age > 52 years old (*n* = 68,118)						
No drink	1.00		1.00		1.00	
Mild	1.13 (1.07–1.20)	<0.001^∗^^†^	1.15 (1.09–1.22)	<0.001^∗^^†^	1.13 (1.07–1.20)	<0.001^∗^^†^
Heavy	0.98 (0.90–1.06)	0.579	1.00 (0.92–1.09)	0.937	1.01 (0.93–1.10)	0.797
Men (*n* = 47,123)						
No drink	1.00		1.00		1.00	
Mild	1.21 (1.13–1.30)	<0.001^∗^^†^	1.28 (1.19–1.38)	<0.001^∗^^†^	1.26 (1.17–1.36)	<0.001^∗^^†^
Heavy	0.92 (0.84–1.02)	0.101	1.02 (0.92–1.13)	0.710	1.02 (0.92–1.13)	0.682
Women (*n* = 87,732)						
No drink	1.00		1.00		1.00	
Mild	1.06 (1.00–1.12)	0.040^∗^^†^	1.11 (1.05–1.18)	<0.001^∗^^†^	1.10 (1.03-1.16)	0.002^∗^^†^
Heavy	0.95 (0.88–1.03)	0.232	1.02 (0.94–1.11)	0.680	1.03 (0.95-1.18)	0.533

^∗^ *P* < 0.05 was considered statistically significant. ^†^Logistic regression model. ^‡^Model 1 was adjusted for age, sex, body mass index (BMI), diabetes mellitus, smoking, alcohol consumption, and nutritional intake. ^§^Model 2 was adjusted for model 1 plus frequency of coffee, green tea, and soft drink.

## Data Availability

Access to data is restricted.

## Supplementary Materials

Supplementary Materials Supplement Table 1 (Table S1): crude and adjusted odds ratios (95% confidence interval) of coffee, green tea, and soft drink intake for periodontitis.

## References

[1] GBD 2015 Disease and Injury Incidence and Prevalence Collaborators (2016). Global, regional, and national incidence, prevalence, and years lived with disability for 310 diseases and injuries, 1990-2015: a systematic analysis for the Global Burden of Disease Study 2015. *The Lancet*.

[2] Kassebaum N. J., Smith A. G. C., Bernabé E. (2017). Global, regional, and national prevalence, incidence, and disability-adjusted life years for oral conditions for 195 countries, 1990-2015: a systematic analysis for the global burden of diseases, injuries, and risk factors. *Journal of Dental Research*.

[3] Nibali L., Di Iorio A., Tu Y.-K., Vieira A. R. (2017). Host genetics role in the pathogenesis of periodontal disease and caries. *Journal of Clinical Periodontology*.

[4] van der Weijden F., Slot D. E. (2011). Oral hygiene in the prevention of periodontal diseases: the evidence. *Periodontology 2000*.

[5] Loe H. (2000). Oral hygiene in the prevention of caries and periodontal disease. *International Dental Journal*.

[6] Levine R. (2012). Obesity and oral disease - a challenge for dentistry. *British Dental Journal*.

[7] Jepsen S., Blanco J., Buchalla W. (2017). Prevention and control of dental caries and periodontal diseases at individual and population level: consensus report of group 3 of joint EFP/ORCA workshop on the boundaries between caries and periodontal diseases. *Journal of Clinical Periodontology*.

[8] Narotzki B., Reznick A. Z., Aizenbud D., Levy Y. (2012). Green tea: a promising natural product in oral health. *Archives of Oral Biology*.

[9] McKay D. L., Blumberg J. B. (2002). The role of tea in human health: an update. *Journal of the American College of Nutrition*.

[10] Venkateswara B., Sirisha K., Chava V. K. (2011). Green tea extract for periodontal health. *Journal of Indian Society of Periodontology*.

[11] Kushiyama M., Shimazaki Y., Murakami M., Yamashita Y. (2009). Relationship between intake of green tea and periodontal disease. *Journal of Periodontology*.

[12] de Almeida J. M., Marques B. M., Novaes V. C. N. (2019). Influence of adjuvant therapy with green tea extract in the treatment of experimental periodontitis. *Archives of Oral Biology*.

[13] de Mejia E. G., Ramirez-Mares M. V. (2014). Impact of caffeine and coffee on our health. *Trends in Endocrinology and Metabolism*.

[14] Han K., Hwang E., Park J. B. (2016). Association between consumption of coffee and the prevalence of periodontitis: The 2008-2010 Korea National Health and Nutrition Examination Survey. *PLoS One*.

[15] Kim H. J., Cho S., Jacobs D. R., Park K. (2014). Instant coffee consumption may be associated with higher risk of metabolic syndrome in Korean adults. *Diabetes Research and Clinical Practice*.

[16] Tsou S. H., Hu S. W., Yang J. J., Yan M., Lin Y. Y. (2019). Potential oral health care agent from coffee against virulence factor of periodontitis. *Nutrients*.

[17] Fiorillo L., Cervino G., Laino L. (2019). Porphyromonas gingivalis, periodontal and systemic implications: a systematic review. *Dentistry Journal*.

[18] Johnson R. K., Appel L. J., Brands M. (2009). Dietary sugars intake and cardiovascular health: a scientific statement from the American Heart Association. *Circulation*.

[19] Bray G. A., Popkin B. M. (2014). Dietary sugar and body weight: have we reached a crisis in the epidemic of obesity and diabetes?: health be damned! Pour on the sugar. *Diabetes Care*.

[20] Menezes C. C., Ribeiro C. C. C., Alves C. M. C. (2019). Soft drink consumption and periodontal status in pregnant women. *Journal of Periodontology*.

[21] Fann J. C., Lai H., Chiu S. Y., Yen A. M., Chen S. L., Chen H. H. (2016). A population-based study on the association between the intake of soft drinks and periodontal disease in Taiwanese adults aged 35-44 years (KCIS no. 33). *Public Health Nutrition*.

[22] Song I. S., Han K., Ko Y., Park Y. G., Ryu J. J., Park J. B. (2016). Associations between the consumption of carbonated beverages and periodontal disease: the 2008-2010 Korea national health and nutrition examination survey. *Medicine*.

[23] Kim Y., Han B.-G., the KoGES group (2017). Cohort profile: the Korean genome and epidemiology study (KoGES) consortium. *International Journal of Epidemiology*.

[24] Ahn Y., Kwon E., Shim J. E. (2007). Validation and reproducibility of food frequency questionnaire for Korean genome epidemiologic study. *European Journal of Clinical Nutrition*.

[25] Byun S. H., Min C., Park I. S. (2020). Increased risk of chronic periodontitis in chronic rhinosinusitis patients: a longitudinal follow-up study using a national health-screening cohort. *Journal of Clinical Medicine*.

[26] Byun S. H., Min C., Kim Y. B. (2020). Analysis of chronic periodontitis in tonsillectomy patients: a longitudinal follow-up study using a national health screening cohort. *Applied Sciences*.

[27] Byun S. H., Lee S., Kang S. H., Choi H. G., Hong S. J. (2020). Cross-sectional analysis of the association between periodontitis and cardiovascular disease using the Korean Genome and Epidemiology Study data. *International Journal of Environmental Research and Public Health*.

[28] Byun S. H., Min C., Hong S. J., Choi H. G., Koh D. H. (2020). Analysis of the relation between periodontitis and chronic gastritis/peptic ulcer: a cross-sectional study using KoGES HEXA data. *International Journal of Environmental Research and Public Health*.

[29] Byun S.-H., Yoo D.-M., Lee J.-W., Choi H.-G. (2020). Analyzing the association between hyperuricemia and periodontitis: a cross-sectional study using KoGES HEXA data. *International Journal of Environmental Research and Public Health*.

[30] Almoznino G., Gal N., Levin L. (2020). Diet practices, body mass index, and oral health-related quality of life in adults with periodontitis- a case-control study. *International Journal of Environmental Research and Public Health*.

[31] Southerland J. H., Taylor G. W., Moss K., Beck J. D., Offenbacher S. (2006). Commonality in chronic inflammatory diseases: periodontitis, diabetes, and coronary artery disease. *Periodontology 2000*.

[32] Hujoel P. (2009). Dietary carbohydrates and dental-systemic diseases. *Journal of Dental Research*.

[33] Sculley D. V., Langley-Evans S. C. (2003). Periodontal disease is associated with lower antioxidant capacity in whole saliva and evidence of increased protein oxidation. *Clinical Science (London, England)*.

[34] Marchetti E., Monaco A., Procaccini L. (2012). Periodontal disease: the influence of metabolic syndrome. *Nutrition & Metabolism*.

[35] Inagaki K., Kurosu Y., Yoshinari N., Noguchi T., Krall E. A., Garcia R. I. (2005). Efficacy of periodontal disease and tooth loss to screen for low bone mineral density in Japanese women. *Calcified Tissue International*.

[36] McGartland C., Robson P. J., Murray L. (2003). Carbonated soft drink consumption and bone mineral density in adolescence: the Northern Ireland Young Hearts project. *Journal of Bone and Mineral Research*.

[37] Tucker K. L., Morita K., Qiao N., Hannan M. T., Cupples L. A., Kiel D. P. (2006). Colas, but not other carbonated beverages, are associated with low bone mineral density in older women: the Framingham Osteoporosis Study. *The American Journal of Clinical Nutrition*.

[38] Hu F. B., Malik V. S. (2010). Sugar-sweetened beverages and risk of obesity and type 2 diabetes: epidemiologic evidence. *Physiology & Behavior*.

[39] Dhingra R., Sullivan L., Jacques P. F. (2007). Soft drink consumption and risk of developing cardiometabolic risk factors and the metabolic syndrome in middle-aged adults in the community. *Circulation*.

[40] de Koning L., Malik V. S., Kellogg M. D., Rimm E. B., Willett W. C., Hu F. B. (2012). Sweetened beverage consumption, incident coronary heart disease, and biomarkers of risk in men. *Circulation*.

[41] Gartenmann S. J., Weydlich Y. V., Steppacher S. L., Heumann C., Attin T., Schmidlin P. R. (2019). The effect of green tea as an adjunct to scaling and root planing in non-surgical periodontitis therapy: a systematic review. *Clinical Oral Investigations*.

[42] Catanzaro D. P., Mena Laura E. E., Cestari T. M. (2018). Green tea prevents vascular disturbs and attenuates periodontal breakdown in long-term hyperglycaemia in T1D rats. *Journal of Clinical Periodontology*.

[43] Taleghani F., Rezvani G., Birjandi M., Valizadeh M. (2018). Impact of green tea intake on clinical improvement in chronic periodontitis: a randomized clinical trial. *Journal of Stomatology, Oral and Maxillofacial Surgery*.

[44] Tanaka K., Miyake Y., Sasaki S. (2008). Beverage consumption and the prevalence of tooth loss in pregnant Japanese women: the Osaka Maternal and Child Health Study. *Fukuoka Igaku Zasshi*.

[45] Machida T., Tomofuji T., Ekuni D. (2014). Severe periodontitis is inversely associated with coffee consumption in the maintenance phase of periodontal treatment. *Nutrients*.

[46] Ng N., Kaye E. K., Garcia R. I. (2014). Coffee consumption and periodontal disease in males. *Journal of Periodontology*.

[47] Mitchell D. C., Knight C. A., Hockenberry J., Teplansky R., Hartman T. J. (2014). Beverage caffeine intakes in the U.S.. *Food and Chemical Toxicology*.

[48] Knight C. A., Knight I., Mitchell D. C. (2006). Beverage caffeine intakes in young children in Canada and the US. *Canadian Journal of Dietetic Practice and Research*.

