# Supplementary material for: Evaluation of the Relationship between Drink Intake and Periodontitis Using KoGES Data
Source: Biomed Res Int. 2021 Mar 16;2021:5545620. doi: 10.1155/2021/5545620 (PMC7990540; doi:10.1155/2021/5545620)
Supplement: Supplementary Materials — Supplement Table 1 (Table S1): crude and adjusted odds ratios (95% confidence interval) of coffee, green tea, and soft drink intake for periodontitis. [file 5545620.f1.docx]

## Supplementary Material

Supplement Table 1(Table S1): Crude and adjusted odds ratios (95% confidence interval) of coffee, green tea, and soft drink intake for periodontitis.

| Characteristics | | | Odds ratio for periodontitis | | | | | |
| --- | --- | --- | --- | --- | --- | --- | --- | --- |
|  |  | | Crude^†^ | *P*-value | Model 1^‡^ | *P*-value | Model 3^§^ | *P*-value |
| Total participants (n= 134,855) | | | | | | | | |
|  | Coffee | |  |  |  |  |  |  |
|  |  | None | 1.00 |  | 1.00 |  | 1.00 |  |
|  |  | Mild | 1.09  (1.02–1.16) | 0.015^*†^ | 1.11  (1.04–1.19) | 0.002^*†^ | 1.05  (0.98–1.12) | 0.190 |
|  |  | Heavy | 0.96  (0.91–1.02) | 0.190 | 0.97  (0.92–1.03) | 0.356 | 1.11  (1.03–1.20) | 0.005^*†^ |
|  | Green tea | |  |  |  |  |  |  |
|  |  | None | 1.00 |  | 1.00 |  | 1.00 |  |
|  |  | Mild | 1.14  (1.08–1.21) | < 0.001^*†^ | 1.14  (1.07–1.20) | < 0.001^*†^ | 0.97  (0.92–1.03) | 0.373 |
|  |  | Heavy | 1.02  (0.98–1.07) | 0.294 | 0.99  (0.95–1.04) | 0.694 | 1.05  (0.96–1.14) | 0.272 |

*^*^* *P* < 0.05 was considered as statistically significant.

^†^ Logistic regression model.

^‡^ Model 1 was adjusted for age, sex, body mass index, diabetes mellitus, smoking, alcohol consumption, and nutritional intake.

^§^ Model 3 was adjusted for model 1 plus frequency of coffee and green tea consumption.
